# Influence of calcium chloride and pH on soluble complex of whey protein‐basil seed gum and xanthan gum

**DOI:** 10.1002/fsn3.2624

**Published:** 2021-10-22

**Authors:** Mozhdeh Sarraf, Sara Naji‐Tabasi, Adel Beig‐babaei

**Affiliations:** ^1^ Department of Food Chemistry Research Institute of Food Science and Technology Mashhad Iran; ^2^ Department of Food Nanotechnology Research Institute of Food Science and Technology Mashhad Iran

**Keywords:** hydrocolloid, ionic strength, rheological behavior, turbidity

## Abstract

Interaction between biopolymers generates different rheological behaviors, which can be effective on the structure of food products. One way to control the polysaccharide–protein interaction is the variation of acidic and ionic strength. In this research, the different amounts of pHs (3–7) and calcium chloride (5–20 mM) were investigated on a soluble complex of whey protein concentrate (WPC) with xanthan gum (XG) and basil seed gum (BSG). The complex characteristic was investigated according to turbidity, viscosity behavior, and electrostatic interactions. The turbidity test showed that WPC:BSG and WPC:XG absorbance increased at pH 3.5 and 4.5, respectively, due to the formation of insoluble complex. pH 6 was the start point of the turbidity increment, which showed the formation of soluble complexes between WPC and polysaccharides. The FTIR analysis confirmed creation of soluble complex at pH 6. The absorbance raised with increasing the molar of CaCl_2_^to^ 10 mM, but no significant difference was observed by turbidity test in the range of CaCl_2_<10 mM. Also, the highest viscosity value was obtained by 10 mM CaCl_2_.

## Graphical abstract

This research paper has been written based on our latest investigation on the creation soluble complex of whey protein concentrate (WPC) with xanthan gum (XG) and basil seed gum (BSG) under different amounts of pHs (3–7) and calcium chloride (5–20 mM). The complex characteristic was investigated according to turbidity, rheological behavior, and electrostatic interactions. The results illustrated although the hydrocolloids had different sources, their functions were approximately similar. The data analysis illustrated that alterations of pH affect soluble and insoluble complexes, and reduced complex viscosity. In addition, the viscosities of complexes had downward trends in the presence of different concentrations of salt. The soluble complex for both hydrocolloids was created in pH 6.

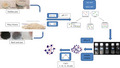

## 1 INTRODUCTION

Interaction of protein and polysaccharides plays a significant role in food structures and their stability in developing the novel formulation. The interaction of macromolecular polymers is a significant issue in the formulation of fabricated products, for instance, the texture of dairy products (Benichou et al., 2007; Corredig et al., 2011; Guzey & McClements, 2006; Naji‐Tab asi et al., 2019; Schmitt et al., 1998; Tolstoguzov, 1997).

Polysaccharides are hydrophilic compounds without surface activity used in the aqueous phase due to the thickening agents to modify the rheological behavior of food products. One of the hydrocolloids which is widely used in food industries is xanthan gum (XG). It is an extracellular anionic polysaccharide with a molecular weight of 10^6^ Da, which is created by *Xanthomonas campestris* (Bertrand & Turgeon, 2007). XG aqueous solutions generate high viscosity and pseudoplastic behavior. They have remarkable efficiency and stability over wide pH, temperature, and salt content ranges surveyed in further research (Murad et al., 2019; Naji et al., 2012). The interaction of XG with other polysaccharides like galactomannans or by cross‐linking in the presence of metal ions formed a gel. The molecular structure prohibits gel formation by itself (Bertrand & Turgeon, 2007). Also, the hydrophobic and electrostatic interaction between XG and protein has been proved. The formed hybrids can provide synergistic effects on the emulsifying and abilities of biopolymers and increasing the steadiness of emulsion droplets against coalescence and flocculation (Benichou et al., 2007).

*Ocimum Basilicum,* commonly known as Basil, belongs to the Lamiaceae family is a one‐year‐old herb with small shoots and a favorable aroma (Dode et al., 2003; Padel et al., 2009). The secondary wall of basil seeds is covered with mucilage (Avlani et al.,). Basil seed gum (BSG) with high molecular weight (2,320 kDa) is used as a stabilizer, fat replacer, controller of ice crystal growth, suspending agent, and emulsion stabilizer in the formulation of pharmaceutical suspensions and food products (Avlani et al.,; Razavi et al., 2011; Razavi & Naji‐Tab asi, 2017; Zeynali et al., 2019). The rheological behavior of BSG affects the improvement of the functional properties of foods (Razavi et al., 2007). Previous research showed that the BSG could create a weak gel in the environment and increase the yield stress, and create a reversible gel with heat (Rafe et al., 2012).

Whey protein is extensively used as a food ingredient due to its useful and nutritious characteristics (Arriaga, 2011). Also, it is applied as an emulsifier and a gelling agent. It is an available and cheap ingredient of by‐products in the dairy industry with powers of thickening and water‐binding capacity. In addition, it is considered as a nutritional compound in the formulation. By adsorption at the emulsion droplet surface, whey proteins stabilize the network by creating an electrostatic barrier against flocculation and coalescence. Whey protein concentrate (WPC) is a type of whey protein that commercially contains 35 to 95% protein. The proteins help in water binding and sometimes add to the gel matrix (Dickinson, 2003; Nayebzadeh et al., 2007). One of the essential features of whey proteins is gelation that can be obtained in different ways. The cold gelation process is one of them. Many factors affect the stability of whey protein emulsions, such as ionic strength, pH, thermal processing, aqueous phase, and emulsifiers (Ye & Taylor, 2009; Zhong et al., 2021). Hydrocolloids‐protein interaction can improve proteins’ functionality. The main factors affecting electrostatic‐driven interactions between proteins and polysaccharides are biopolymer ration, biopolymer concentration, ionic strength, and pH (Hosseini et al., 2013; Khoshmanzar et al., 2013; Neirynck et al., 2007; Schmitt & Turgeon, 2011; Yang et al., 2021).

pH is one of the significant parameters effective in interacting with protein‐polysaccharide complexes, and varying electrostatic interaction creates soluble complexes or insoluble ones (Raei et al., 2018; Weinbreck et al., 2003a; Ye, 2008). Proteins carry positive and negative charges on the medium pH and the electrical charges toward various amino acids in the protein molecules and their ionization mode in different pHs (Ghosh & Bandyopadhyay, 2012). The other important factor in the interaction between two biopolymers is strength of ionic compounds like NaCl and CaCl_2_. They decrease interaction between protein molecules and raise aggregation. It is also involved in the formation of cross‐linking between unfolded protein chains resulting in network formation (Mulvihill & Kinsella, 1988). Ca^2+^ ions use as chelating agents in xanthan chains and other charged polysaccharides (Patel et al., 2020).

The purpose of the study was to investigate the interactions of WPC: XG and WPC: BSG at different pHs and concentrations of calcium. The other target was a comparison of function between two gums used that come from two various sources.

## 2 MATERIALS AND METHODS

### 2.1 Material

Basil seeds were purchased from a local market (Mashhad, Iran). Xanthan gum (Sigma, Germany) and whey protein concentrated powder (WPC) (Westland, New Zealand) were purchased. In addition, sodium azide, hydrogen chloride (HCl), sodium hydroxide (NaOH), and calcium chloride (CaCl_2_) were supplied from Merck Co. (German). Deionized water was used to prepare all solutions.

### 2.2 Extraction of Basil Seed Gum

The extraction of BSG was done according to the procedure of Naji‐Tabasi et al. 2016 (Razavi et al., 2011). In brief, the distilled water was added to seeds in a ratio of 20:1. They were mixed for 20 min at 68°C, after which, the separation of mucilage from seeds was conducted by a lab extractor by scraping technique. Then the mucilage was mixed with ethanol 96% (1:3) to precipitate the polysaccharide. After dissolving in distilled water, the extraction was dried in an air‐forced oven (Memert‐UF55: German) at 40°C.

### 2.3 Preparation of Protein: polysaccharide complexes

BSG and XG stock dispersions (0.1% w/w) and WPC (2% w/w) were prepared with deionized water. They were stirred for 2 hr and put in the refrigerator for 24 hr to complete their hydration. 0.02% (w/w) sodium azide was added to prevent bacterial growth. The mixtures of polysaccharide‐protein were prepared in a ratio of 1:1. The solutions were stirred for 30 min to mix completely. Before carrying out experiments, their pHs were justified with HCl and NaOH 0.1 N in the range of 3–7 by a pH meter (Metrohm, Switzerland) (Khalesi et al., 2016; Naji‐Tab asi & Razavi, 2017b).

To consider the influence of calcium ion concentration on the samples, the different concentrations of CaCl_2_ (5–20 mM) were added to the selected treatment (soluble complex) of the previous step according to analysis.

### 2.4 Determination of Turbidimetric

The turbidity of solutions was determined using a DR‐500 UV‐visible spectrophotometer (Hach, Germany) at a wavelength of 633 nm (Naji‐Tab asi et al., 2019). All measurements were done in the ambient room.

### 2.5 Determination of ζ‐potentials

Electrostatic interactions were measured to investigate surface density changes around the newly formed hybrids between protein and polysaccharide molecules. ζ‐potential measurements were performed using the electrophoretic light scattering of Zeta Potential (Zeta Compact CAD, France) at 25°C (Naji‐Tab asi et al., 2019). Each treatment is measured three times.

### 2.6 Determination of rheological behaviour

Viscosity properties were investigated with a viscometer (Brookfield, LV DVIII Ultra, USA) in the shear rate range of 0.1–100 s^‐1^ at 25°C by a heating circulator (Julabo, Model F‐12‐MC, Germany). A computer‐controlled program was used to shear solutions ( Naji‐Tab asi & Razavi, 2017a).

### 2.7 Determination of FT‐IR

The functional groups of protein, gums, and soluble complexes of WPC: BSG, and WPC: XG were evaluated in powder form by Fourier Transform Infrared Spectrometer (Thermo Nicolet AVATAR 370, USA) between wavelength 400 to 4,000 cm^‐1^ (Faria et al., 2011; Naji‐Tab asi et al., 2019).

### 2.8 Statistical analyses

The complete randomized factorial design was used for statistical analysis. The data were analyzed by SPSS (ver.11.0) according to ANOVA. The significant difference was obtained by Duncan's multiple range tests at level 95%. Three replications were used for treatments. The curved plotted by the software Excel 2013.

## 3 RESULTS AND DISCUSSIONS

### 3.1 The effects of pH on the solution turbidity

**FIGURE 1:**
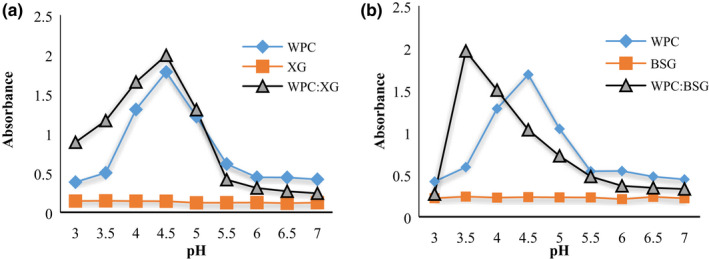
Turbidity measurement of WPC, XG, WPC: XG (a), and WPC, BSG, WPC: BSG (b) as a function of pH (3–7)

Figure 1 shows the turbidity of the system as a function of pH. The turbidity of hydrocolloid dispersions was stable in pH of 3–7. The reason for the turbidity of polysaccharides is high molecular weights; a polymer with a high molecular weight gives a higher adsorption density (Garcia Vidal, 2013).

Results analysis of protein turbidity in the absence of polysaccharides showed that WPC absorbance raised with increasing pH toward 4.5. A protein and an anionic polysaccharide carry opposite net charges at pH<IP of the protein, resulting in a maximum electrostatic attraction (Xia & Dubin,). The major peak of WPC turbidity was at pH ~4.5 that it shows IP of protein (Gulzar, 2011). After this, the increasing pH led to a decrease in turbidity. The electrostatic force is an essential driving force in the solution and effects on aggregation and agglomeration (Sun et al., 2008).

Insoluble complexes are generated by binding anionic polysaccharides to cationic proteins at pH<pI. The charge of the protein changes from negative to positive when the pH decreases to lower than the *pI* of protein. The binding initially causes charge neutralization, which leads to the formation of an insoluble aggregation complex. However, binding anionic polysaccharides with protein at pH>pI resulted in forming soluble complexes (Ghosh & Bandyopadhyay, 2012; Schmitt et al., 1998).

The most absorbance of WPC:BSG and WPC:XG solutions were observed at pH 3.5 and 4.5, respectively, because of the insoluble complexes formation. Complexes of WPC:BSG and WPC :XG were generated between oppositely charged electrolytes. That means the most interaction occurred, and the final charge of the system is weak. The system turbidity declined with decreasing pH value sharply in complexes of BSG (3.5>) and gradually in complexes of XG (4.5>), which may be related to complex dissolution. The results agreed with the other reports (Hefnawy & Ramadan, 2011; Malhotra & Coupland, 2004; Raoufi et al., 2017).

In pH values more than *PI*, protein and polysaccharide net charges are the same and cause soluble complexes. The complexes have enough negative to be soluble. Because fewer proteins can interact with the polysaccharide as fewer charged moieties are accessible (Naji‐Tabasi et al., 2020). There were no significant oscillations in both the complexes diagrams from pH 6–7 (Figure 1 a,b). As the pH decreased to 6, the system's turbidity gradually started to increase. The start point of increment showed the formation of soluble complexes between WPC and polysaccharides.

### 3.2 The effects of pH on the ζ‐potentials of solution

**FIGURE 2:**
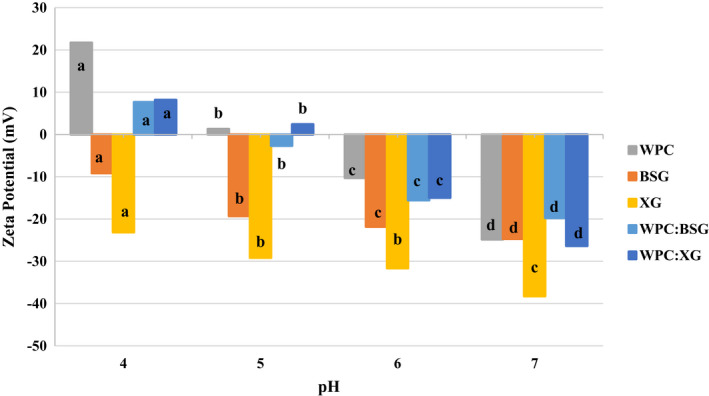
Zeta Potential of WPC, BSG, XG, WPC:BSG, and WPC:BSG based on changing pH (4–7)

The ζ‐potential of solutions at different pH values from 4 to 7 is shown in Figure 2. It showed pH~5 is the critical point for WPC. The ζ‐potential of WPC was the most positive in pH 3. The pH values altered from +21.72 to −24.82 as the pH increased from 4 to 7.

The ζ‐potential value of BSG was negative between pH 4 ‐7 . The ζ‐potential of BSG was the most negative in pH 4 (Figure 2). Also, the ζ‐potential of XG was negative in all pH.

ζ‐potential value of complexes illustrated a decreasing trend of ζ‐potential value with addition polysaccharide to the WPC solution due to raising the number of carboxyl groups on the side chains (Shiroodi, 2014). Therefore, the groups reduced at pH>PI of WPC, following, ζ‐potential of mixes trend toward negative charges (Raoufi et al., 2017).

When the pH decreased from 7 to 4, the ζ‐potential of complexes were more positive than pure hydrocolloid solutions that indicated the electrostatic complexes’ formation with positive charges (Raoufi et al., 2017; Shiroodi, 2014). According to Figure 2, pH ~6 can be considered as the start point of the soluble complex. Both, protein and polysaccharides had negative charge, but they created complexes. According to the results of electrostatic interactions and turbidity, the formation of insoluble WPC:XG and WPG:BSG complexes occurred at pH_C_ 4.5 and 3.5, respectively. Shiroodi (2014) reported that the formation of soluble WPI and XCHC (xanthan curdlan hydrogel complex) complexes is attributed to the interaction between oppositely charged biopolymers, the presence of positive surface charges of protein, and negatively charged XCHC (Shiroodi, 2014).

### 3.3 The effect of pH on rheological behavior

**FIGURE 3:**
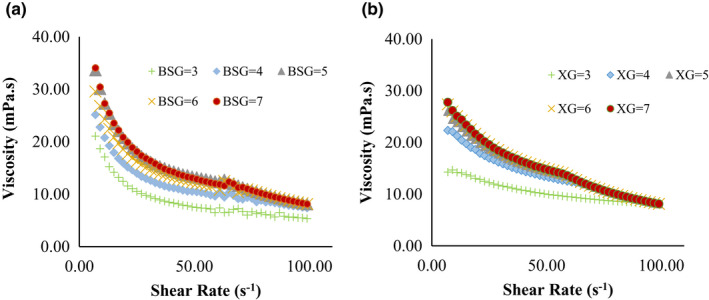
Apparent viscosity of BSG (a) XG (b) solutions based on shear rate (0–100)

**FIGURE 4:**
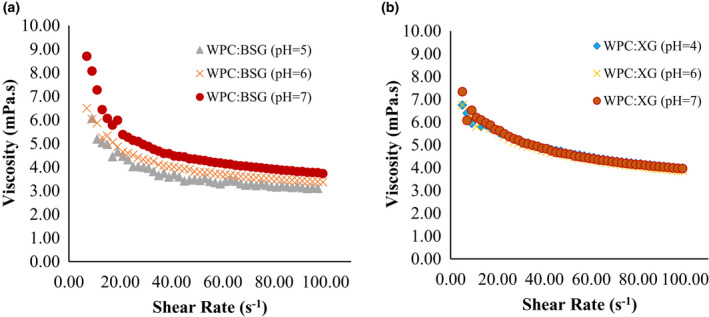
Apparent viscosity of WPC:BSG (a), and WPC: XG (b) complex (0–100 s^‐1^)

As a result, the flow curve of shear rate and apparent viscosity at different treatments are presented in Figures 3 and 4.

The viscosities of BSG and XG decreased with increasing shear rate, which indicates a shear‐thinning behavior (Figure 4a, b). This behavior has been reported in previous studies (Naji et al., 2012; Naji‐Tab asi & Razavi, 2017b). In addition, viscosities declined with the change in the pH toward more H^+^. The maximum viscosity was gained around pH 7, where the shape of hydrocolloid chains is close to the rod conformational state (Achi & Okolo, 2004). Also, the viscosity of BSG had a higher viscosity than XG at pH 7. In general, carboxyl groups are gradually ionized, the coils are expanded with raising pH because of electrostatic repulsion between the functional groups in hydrocolloids, which causes intermolecular binding, and the viscosity of solution increased (Feng et al., 2007).

According to the results obtained from hydrocolloid solutions, predictable results were shown WPC:BSG and WPC:XG complexes (Figure 4a,b). A decrease in viscosity was also observed after complex formation. Therefore, the coacervates are generated from electrostatic interactions between the WPC and hydrocolloids at low pHs (Bastos et al., 2010). In both gums, the viscosity trends were decreasing, although the WPC:BSG viscosity was still higher than the WPC:XG viscosity at all pHs. The viscosity decreased noticeably after creation complexes of WPC:BSG, and the viscosities decreased in all complexes by decreasing pH. There was no difference between pH 5 and 6. WPC:XG complexes in all pHs had shear‐thinning property and there was no difference between the viscosities in different pH values.

### 3.4 FTIR analysis

**FIGURE 5:**
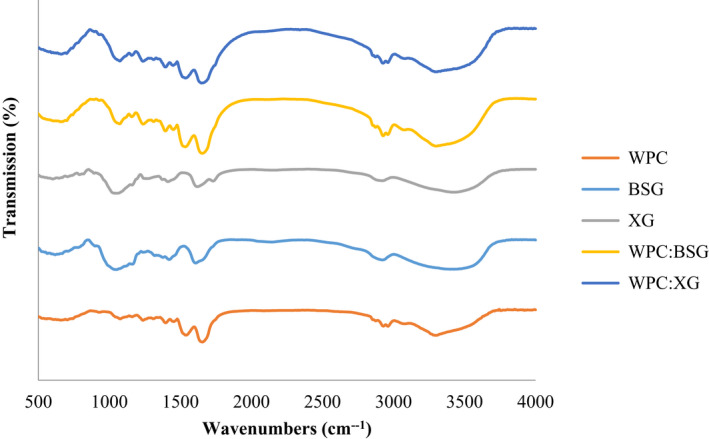
FTIR spectra of WPC (a) BSG (b) XG (c) WPC:BSG (d) and WPC:XG (e) in pH 6 (25°C)

FTIR spectrum analysis was conducted on protein and hydrocolloids powders, and dried WPC: BSG, and WPC: XG complex in pH =6 (Figure 5) to be sure about creating soluble complex in these pH value.

As Figure 5 shows, the absorption of WPC at 3,299 cm^‐1^ corresponds to stretching vibrations of OH linked to NH_2_. Bands ~ 2,900 cm^‐1^ are related to CH_2_ groups. The absorption at the wavenumber 1640 and 1538 cm^‐1^ are a distinct band of primary amide of the group (‐CO‐NH_2_) and secondary amide of the group (‐CO‐NH), respectively, that well were characterized in the whey proteins. Also, ~ 900–1400 cm^‐1^ is related to CO, CC, and COH groups. The most significant peak was the corresponding absorption of the crystal (~2000 cm^‐1^) (Gbassi et al., 2012).

The most substantial adsorption peaks at 3,435 and 3,427 cm^‐1^ in XG and BSG, respectively, show the axial deformation of hydrogen‐bonded OH. The graphs of BSG and XG showed that the absorption at the wavenumber ~1600–1654 cm^‐1^ is related to free carboxylate groups. The absorptions at wavenumber 1,400 and 1,600 cm^‐1^ are assigned to C–OO symmetric and asymmetric stretching, respectively, which confirmed the presence of uronic acid. The peak found at ~1,300 cm^−1^ showed the deflection angle of CH. The peak located at ~1,000 cm^−1^ showed axial deformation of CO (Faria et al., 2011; Li et al., 2016; Mohsin et al., 2018; Moosavi‐Nasab et al., 2010; Naji‐Tab asi et al., 2019).

As Fig.5 is shown, a peak in ~1073 cm‐1 of the complexes of WPC:BSG and WPC:XG is observed , which was reduced by increasing hydrocolloids that be attributed to the non‐esterified carboxyl group(Gnanasambandam & Proctor, 1999).

The FTIR result of the isolated soy protein and BSG combination showed that BSG altered the infrared spectrum of the protein. The frequencies of the bands remained constant in amide I and II regions with adding BSG. In addition, other peaks were observed in the range of 1160–1600 cm^‐1^, which are related to the interaction between amide groups of protein and hydrocolloid (Jung, 2000). There was an individual peak around 2,800 cm^‐1^, which indicates the OH stretching of water molecules. When complex polymers are thermodynamically compatible and intermolecular interactions predominate, the FTIR spectra of the compounds differ from those of the constituent polymers.

On the other hand, a combination of two incompatible polymers produces the FTIR spectrum in which the two polymers are superimposed. The FTIR result strongly indicates that the two polymers are thermodynamically incompatible because the amide I peak increases in the protein and hydrocolloids. When blended polymers are thermodynamically compatible and intermolecular interactions prevail, the resultant FTIR spectra of the combinations differ from those of the component polymers (Rafe & Razavi, 2015). Based on the test results on different pHs, interaction of soluble complex was adjusted at pH =6 (Pilevaran et al., 2021).

### 3.5 Effect of Calcium Chloride on the solution turbidity in pH =6

The reason for adding calcium ions to the solution is reducing repulsive negative charges between protein molecules and increasing the tendency toward aggregation. It also affects the generation of cross‐linking between unfolded protein chains network aggregation (Rafe et al., 2013).

**FIGURE 6:**
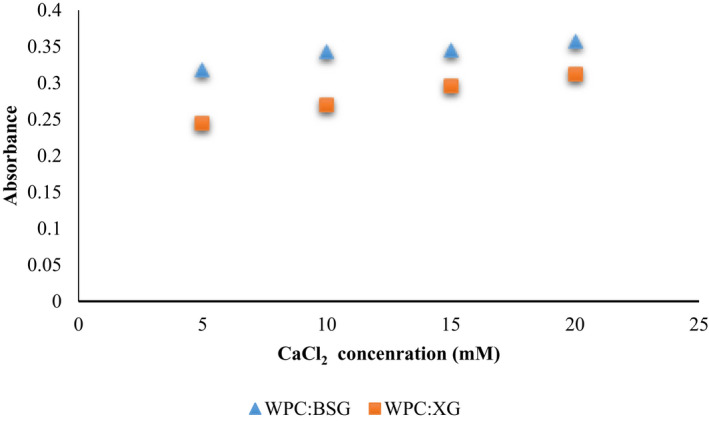
Turbidity measurement of WPC:XG, and WPC:BSG: different concentrations of CaCl_2_ (5–20 mM) in pH =6

Therefore, the effect of Ca^2+^ ion on WPC:XG and WPC:BSG turbid were studied at different concentrations of 5,10,15, and 20 mM in pH =6 that are shown in Figure 6.

As can be seen, the turbidity increased in both of the mixtures with increasing concentration of CaCl_2_. The absorbance of BSG:WPC and XG:WPC in 5 mM were 0.318 and 0.245, respectively. But the increase value of turbidity decreased with increasing Ca^2+^ concentration and absorbance of BSG:WPC and XG:WPC showed 0.357 and 0.312, respectively. Although the amount of absorbance in WPC:BSG was more than the mix of WPC:XG, and the turbidity was dependent on the concentrations of salt; it was well known that the turbidity of the solution increased with calcium level up to 10 mM. The strength of whey protein gels increased(Kuhn & Foegeding, 1991; Mulvihill & Kinsella, 1988). The presence of salt concentrations in solution affects gelation and texture profile because of electrostatic interactions with the negatively charged and unfolded protein molecules (Ju & Kilara, 1998).

### 3.6 The effects of ca^+^ ion on rheological behavior of solutions in pH =6

**FIGURE 7:**
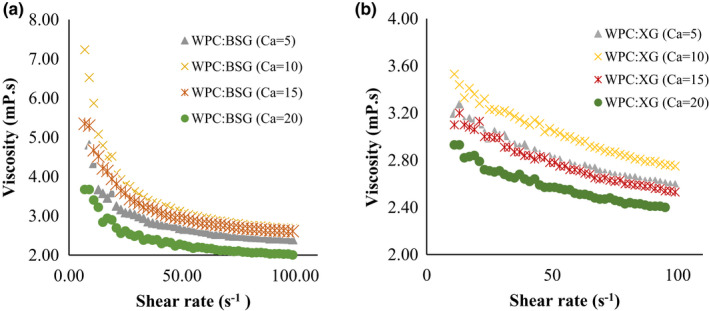
Apparent viscosity of WPC:BSG (a), and WPC:XG (b): different concentrations of *CaCl*_2_(5–20 mM) in pH =6

The rheological properties of WPC:BSG and WPC:XG complex (pH 6) at different concentrations of CaCl_2_ was shown in Figure 7. The results showed a shear‐thinning behavior in the presence of calcium on all samples.

As displayed in Figure 7a, at the low shear rate, the highest viscosity was observed in 10‐mM. However, it decreased by raising salt to 15 and 20 mM. The increase of salt concentration usually led to generate a complex with compact structure. Also, the biopolymer concentration continuously declined with the rise of salt concentration in coacervates, which was related to the electrostatic interaction between protein, and polysaccharide (Wang et al., 2007). The viscosity of WPC:BSG decreased in shear rate from 10 to 20 s^‐1^, and it was approximately stable until 100 s^‐1^ (Figure 7a). The most and least viscosity were observed at 10 and 20 mM CaCl_2_, respectively.

About the trend of WPC:XG complex, at first, the viscosity was at the highest point, and then it decreased and continued stably (Figure 7b), which shows shear‐thinning behavior in all complexes. By comparing the viscosity of both hydrocolloids, BSG generated a complex with more viscosity than XG. Also, screening the charge is influential on the formation of the complex due to the decreasing interaction, while the results showed it is less at low concentrations (Weinbreck et al., 2003b; Ye et al., 2006).

The presence of salt reduced the viscosity of solutions at different pHs As the pH increases, the viscosity decreases due to the molecular cross‐linking between hydrocolloids such as xanthan and calcium ions (Hemmatzadeh et al., 2011).

## 4 CONCLUSION

In the research, the effect of different calcium concentrations and pHs on interactions of WPC:XG and WPC:BSG were investigated. The results showed soluble or insoluble complexes, dependent on the pH, can be established. Insoluble complexes were generated below the isoelectric point of WPC, where the protein and polysaccharides had opposite electrical charges. Soluble complexes were also created at pH =5.5–6, where hydrocolloids were negatively charged, and whey protein had a slight net charge (due to the positively charged patches on the whey protein surface). The FTIR analysis confirmed creation of soluble complex at pH 6. The addition of gums shifted the zeta potential of complex toward the negative charge. Also, the hydrocolloids increased the viscosity of system, and result in shear‐thinning behavior. Higher concentrations of calcium affected the rheological properties of the complexes. But, there was no significant difference in the range of 10–20 mM CaCl2.

## CONFLICT OF INTEREST

The authors declare that they do not have any conflict of interest.

## AUTHOR CONTRIBUTIONS

**Mozhdeh Sarraf:** Formal analysis (equal); Funding acquisition (equal); Investigation (equal); Writing‐original draft (equal). **Sara Naji Tabasi:** Project administration (equal); Supervision (equal); Validation (equal); Writing‐review & editing (equal). **Adel Beig‐babaei:** Supervision (equal); Validation (equal); Writing‐review & editing (equal).

## Data Availability

The data that support the findings of this study are available from the corresponding author upon reasonable request.
